# Recent Advance in Applications of Proteomics Technologies on Traditional Chinese Medicine Research

**DOI:** 10.1155/2015/983139

**Published:** 2015-10-19

**Authors:** Qing Ji, Fangshi Zhu, Xuan Liu, Qi Li, Shi-bing Su

**Affiliations:** ^1^Research Center for Traditional Chinese Medicine Complexity System, Shanghai University of Traditional Chinese Medicine, Shanghai 201203, China; ^2^Department of Medical Oncology, Shuguang Hospital, Shanghai University of Traditional Chinese Medicine, Shanghai 201203, China; ^3^Jiangsu Provincial Academy of Traditional Chinese Medicine, Nanjing 210028, China

## Abstract

Proteomics technology, a major component of system biology, has gained comprehensive attention in the area of medical diagnosis, drug development, and mechanism research. On the holistic and systemic theory, proteomics has a convergence with traditional Chinese medicine (TCM). In this review, we discussed the applications of proteomic technologies in diseases-TCM syndrome combination researches. We also introduced the proteomic studies on the *in vivo* and *in vitro* effects and underlying mechanisms of TCM treatments using Chinese herbal medicine (CHM), Chinese herbal formula (CHF), and acupuncture. Furthermore, the combined studies of proteomics with other “-omics” technologies in TCM were also discussed. In summary, this report presents an overview of the recent advances in the application of proteomic technologies in TCM studies and sheds a light on the future global and further research on TCM.

## 1. Introduction

Traditional Chinese medicine (TCM), emphasizing most importantly the holistic concept, has been applied in the diagnosis, treatment, and prevention of illnesses in China and other Asian countries for more than 3000 years. Because of the complexity of the concept, the technology limitations, and the current investigation methodology, TCM diagnosis and treatment lack objective evaluation, and the essence and the mechanisms of TCM theory remain unclear.

Since proteins are the major executers of biological information, proteomic analysis provides a direct reflection of gene expression. Generally, proteomics is defined as the genome-scale analysis of protein abundance, structure, localization, modification, and activity. Currently, as a major component of systems biology, proteomics has gained comprehensive attention in the field of medical diagnosis, drug development, and mechanism studies [1, 2]. Proteomics technology is an important research tool for elucidating the differential expressions of proteins in peripheral body fluids, cells, tissues, blood, and urine samples [3]. Blood and urine are the most widely used specimens because their molecular compositions fluctuate in response to the dynamic physiological and pathological conditions of the body. Technically, proteomic analysis requires the combination of several technologies, that is, protein processing and separation such as two-dimensional polyacrylamide gel electrophoresis (2DE), high-performance liquid chromatography (HPLC), mass spectrography (MS) such as MALDI-TOF-MS, SELDI-TOF-MS, and MS/MS, isobaric tags for relative and absolute quantification- (iTRAQ-) based quantitative proteomic analysis, and bioinformatics [4].

TCM diagnosis and therapy depend on the intuition and experience of the TCM theory trained physicians. Compared with biomolecular science and western medicine, TCM appears to be nonobjective and lacking accuracy and reproducibility. In accordance with the holistic and systemic theory, proteomics has a convergence with TCM and can overcome biases in TCM research. Proteomics can be helpful in exploring the scientific connotation of TCM and the modernization of Chinese herbal medicine (CHM). First, proteomics could be used to characterize the differential expression profiles between healthy individuals and patients with different TCM syndromes. For example, apolipoprotein A1 and apolipoprotein A4 expression levels analyzed by plasma proteomics were found to be the potential diagnostic and prognostic markers for chronic viral hepatitis B (CHB) with damp-heat retention in Middle-Jiao syndrome (DRMS) [5]. Similar findings [6–10] can discover other molecular markers of TCM syndromes in clinical applications.

Secondly, proteomics can help discover molecular targets, develop new bioactive compounds, and elucidate the underlying mechanisms of TCM treatment. For instance, a recent proteomic study showed that Tianma promoted neuroregenerative processes by inhibiting stress-related proteins and mobilizing neuroprotective genes such as Nucleoredoxin (Nxn), Drebrin-like protein (Dbnl), Ki67 protein, and Baxin mouse N2a cells [11]. This and other similar proteomic studies provide important insights into the molecular mechanisms underlining the beneficial effects of TCM treatments [12–15].

**Figure 1:**
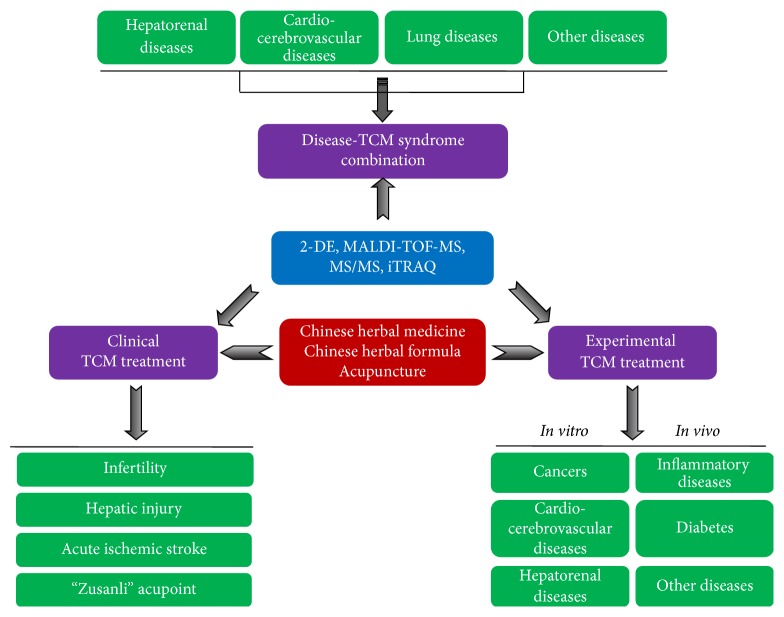
Scheme of the overview of the recent advance in the applications of proteomics technologies on traditional Chinese medicine research.

In this report, we reviewed the current proteomic approaches in TCM research including clinical TCM diagnosis and treatment and* in vitro* and* in vivo* mechanistic studies and shed light on future utilization of proteomics for TCM research (Figure 1).

## 2. Proteomics Studies on Disease-TCM Syndrome Combination

TCM syndrome, also called ZHENG in Chinese, is a profile of clinical symptoms and signs, which reflect the essence of pathological changes in the occurrence and development of diseases, provide great insights in understanding the human homeostasis, and guide specific TCM treatments. TCM syndrome differentiation, that is, the diagnosis of TCM syndrome, is to differentiate diseases by analyzing the information of each patient, for example, patients' symptoms and physical status, which were collected by four diagnostic methods: inspection, auscultation and olfaction, inquiry, and palpation [16]. Currently, although the applications of proteomic technologies in TCM are much less common than those in western medicine, clinical TCM studies using proteomic technologies have already achieved some great successes, with several biomarkers and the mechanisms of TCM syndrome differentiation being discovered in various diseases, such as hepatorenal, cardiocerebrovascular, and lung diseases.

### 2.1. Hepatorenal Diseases and TCM Syndromes

In a chronic hepatorenal diseases-TCM syndrome study, Wei et al. [5] investigated the plasma proteomics of chronic viral hepatitis B (CHB) of damp-heat retention in the Middle-Jiao syndrome (DRMS) using 2DE and MS technologies; they found apolipoproteins A1 and A4 as the diagnostic and prognostic markers or treatment targets. Liu et al. demonstrated that immunoglobulin J-chains protein could be a new biomarker for the diagnosis of different TCM syndromes in CHB [6]. Song et al. [7] established diagnosis models of excess syndrome and deficiency syndrome in CHB by SELDI-based protein chip analysis. Using MALDI-TOF-MS technology, Zhou et al. [8] set up diagnosis models of Spleen-Qi asthenia syndrome, Liver-Kidney Yin deficiency syndrome, and blood stasis syndrome in hepatitis B cirrhosis (HBC).

In addition, Hao et al. [9, 10] established a predictive model for clinical typing of chronic renal failure (CRF), screened for protein markers in urine samples of CRF patients with TCM damp syndrome (CMDS), and illustrated that urine protein biomarkers reflected different biological features of CRF with different TCM syndromes. For example, the levels of* m/z* 1674.53 and* m/z* 1952.7, two differentially expressed proteins, were elevated in Liver-Kidney Yin deficiency but lowered in Spleen-Kidney Qi deficiency, Spleen-Kidney Qi-Yin deficiency, Spleen-Kidney Yang deficiency, and Yin-Yang deficiency.* m/z* 2305.78 and* m/z* 4262.02, another two differentially expressed proteins, were expressed less in Liver-Kidney Yin but more in Spleen-Kidney Qi deficiency, Spleen-Kidney Qi-Yin deficiency, Spleen-Kidney Yang deficiency, and Yin-Yang deficiency.

### 2.2. Cardiocerebrovascular Diseases and TCM Syndromes

In a cardiocerebrovascular diseases-TCM syndrome research, Chu et al. [17, 18] demonstrated that the differentially expressed proteins, such as 9334.958* m/z* (increased), 9280.191* m/z* (decreased), 8030.794* m/z* (increased), and 2941.551* m/z* (increased), might be potential biomarkers of abundant phlegm-dampness syndrome (PDS) and liver-gallbladder dampness-heat syndrome (LGDHS) in hypertension patients.

Song et al. [19] studied correlation between the states of Zang-Fu organs and the levels of plasma biomarker proteins and found differential plasma protein profiles in hyperlipidemia and atherosclerosis of different patterns of phlegm stasis syndrome and blood stagnation syndrome. For example, the levels of albumin, adrenomedullin binding protein precursor, and haptoglobin precursor in patients with phlegm syndrome were different from those in the patients with blood stagnation syndrome and also correlated with kidney-Qi deficiency and heart-Qi deficiency, while the complement component C4 is independent of the deficient Zang-Fu organs. Zhao et al. [20] uncovered common proteomic characteristics in unstable angina with Qi deficiency and blood stasis syndrome (QBS) and phlegm stasis cross-blocking syndromes (PSS), indicating a correlation of these proteins with inflammatory reaction and metabolic disturbance. For instance, actin was found only expressed in Qi deficiency blood stasis syndrome (QDBS), while FN, ApoH, and ANXA6 are highly expressed in QDBS. Wang et al. [21] found that energy metabolism and myocardial structural injury associated proteins, isocitrate dehydrogenase 3 (NAD+) alpha, NADH dehydrogenase (NAD) Fe-S protein 1, chain A, heat shock protein 27 (HSP27), and oxidoreductase (NAD-binding protein), may be biomarkers for the diagnosis of chronic myocardial ischemia with QBS.

### 2.3. Lung Diseases and TCM Syndromes

Using proteomic technology, Liu et al. [22] established a diagnostic serum proteomic model for the three TCM syndromes in tuberculosis (TB), and ApoC-III was identified as a potential biomarker for TCM syndrome differentiation in TB. By combining SELDI-TOF-MS techniques with a decision tree model, nine upregulated and six downregulated proteins were identified in lung cancer patients with Qi deficiency syndrome and phlegm and blood stasis syndrome. Two candidate protein peaks, 2284.97* m/z*, were selected to establish a predictive model, which can be applied in the TCM syndrome differentiation in lung cancer [23].

Other studies have also provided the evidence of syndrome differentiation using proteomic technologies in chronic stomach disease [24], myasthenia gravis [25], and systemic lupus erythematosus [26]. All these studies suggested that the rapid growth of proteomics has made it possible for the integration of diseases-TCM syndrome with modern technology, thereby supplying the diagnostic or prognostic markers for TCM syndrome differentiation, as well as TCM therapy targets.

## 3. Proteomic Studies of Clinical TCM Treatments Using Chinese Herbal Medicine (CHM), Chinese Herbal Formula (CHF), and Acupuncture

TCM treatment is based on the holistic characterization of patients' disease status, which is diagnosed to certain TCM syndrome type. Most of the TCM therapeutic methods, CHM, CHF, or acupuncture, provide modern medicine with a collection of complementary remedies for disease treatment and health maintenance. Although some of CHM, CHF, or acupuncture methods are known to have beneficial effects on the diseases, their therapeutic efficacy could not be well evaluated. Recently, proteomic technologies have been utilized to measure the therapeutic effect of TCM treatments [74].

Lian et al. [75] demonstrated that the favorable effects of Liuwei Dihuang granule (LDG) on infertility of women with Kidney-yin deficiency syndrome might be through regulating the expression levels of retinol binding protein 4, transthyretin, apolipoprotein, and complement C4-B, all being associated with HPT axis, lipid metabolism, estrogen level, and cellular immunity, and activation function of complement system-pathways might be the actionable targets for the treatment of infertility with LDG. To identify the drug targets of TCM formulae Yin-Chen-Hao-Tang (YCHT), which was used to treat hepatic injury, Sun et al. [76] performed 2DE and MALDI-TOF/TOF-MS analysis and found that YCHT modulated the expression levels of several proteins, that is, zinc finger protein 407, haptoglobin, transthyretin, and vitamin D-binding protein, all being involved in metabolism, energy generation, chaperone, antioxidation, signal transduction, protein folding, and apoptosis. Pan et al. [77] investigated the effects of acupuncture on serum protein levels in a total of 35 acute ischemic stroke (IS) patients, with the acupuncture treatment being performed on eight acupuncture points once a day for 10 consecutive days. After acupuncture, SerpinG1 protein expression in patients' serum was downregulated while those of gelsolin, complement component I, C3, C4B, and beta-2-glycoprotein I proteins were upregulated. iTRAQ-based quantitative proteomics was performed to identify key proteins in the blood sample for acupuncture at “Zusanli” acupoint (ST-36) in patients, and a total of seven related proteins were identified. These proteins, aldolase A protein, hCG2008184, ATP synthase, ATP5A1 protein, and hexokinase type 1, were involved in the regulation of multiple metabolism pathways, which may help elucidate the action mechanism of ST-36 acupuncture [78].

Although proteomic studies in clinical TCM medicine have been successful, such studies in clinical TCM treatment still remain few. Ideally, proteomic studies should be conducted in both the diagnosis of a TCM syndrome and its corresponding treatment including CHM, CHF, and acupuncture. A large number of experimental proteomic studies are currently under way to uncover the molecular mechanisms of TCM treatment.

## 4. Proteomic Studies on Mechanisms of TCM Treatments* In Vitro* and* In Vivo*

The complex nature of TCM determines that a thorough investigation on the mechanisms and physical basis of TCM will not be so easy. Luckily, development of modern biotechnologies is constantly providing novel and powerful tools. Proteomic technologies can reveal statistically significant changes in the levels of proteins, identify novel target molecules, and provide clues for the underlying mechanisms of TCM treatments. Herein, we will focus mainly on the application of proteomics in the research of TCM treatment using* in vitro* and* in vivo* models over the past years [79].

### 4.1. Evaluation of TCM Treatment* In Vitro*

Proteomic technologies can be applied to screen target molecules and explore the effective mechanisms of TCM treatments in various cell lines originated from various diseases, such as cancers and cardiocerebrovascular and inflammatory diseases. TCM treatment includes CHM, CHM compounds, and CHF. As shown in Table 1, proteomics is often applied to investigate the change of proteins and of various related signaling pathways in TCM treatments* in vitro*.

#### 4.1.1. Cancers

In cervical carcinoma, Cui et al. [27] tested the cytotoxicity of 9,11-dehydroergosterol peroxide (DHEP) isolated from* Ganoderma lucidum* on HeLa cells and revealed that Stathmin 1 might be a target of DHEP. Yue et al. [28] found that triterpenes from* Ganoderma lucidum* extract targets interleukin-17E, eukaryotic translation initiation factor 5A, peroxiredoxin-2, and ubiquilin-2, which are involved in cell proliferation, carcinogenesis, and oxidative stress. In addition, Pan et al. [29, 30] found by proteomics that Tanshinone IIA had cytotoxic activity against HeLa cells via regulating the expression of proteins involved in apoptotic processes, spindle assembly, and p53 activation.

In hepatocellular carcinoma, through proteomic analysis, Fu et al. [31] discovered that 1,3,6,7-tetrahydroxyxanthone (TTA) effectively induced apoptosis of HepG2 cells through upregulating the expression levels of P16 and 14-3-3*σ* protein while downregulating that of *β*-tubulin. Fu et al. [32] demonstrated that 1,3,5-trihydroxy-13,13-dimethyl-2H-pyran [7,6-b] xanthone promoted mitochondrial apoptosis of HepG2 cells via mediating the heat shock protein 27.

In colorectal adenocarcinoma, Huang et al. [33] found that Baicalein inhibited colorectal cancer DLD1 cell proliferation and reduced reactive oxygen species (ROS) by upregulating the levels of peroxiredoxin-6 (PRDX6). Liu et al. [34] discovered that 14-3-3 epsilon, a cell cycle- and apoptosis-related protein, was affected (including cleavage and perinuclear translocation) in colon cancer SW480 cells treated with Triptolide.

In gastric adenocarcinoma, Lin et al. [35] revealed that Tanshinone IIA suppressed gastric cancer AGS cell growth by blocking glucose metabolism via the downregulation of the levels of intracellular ATP, glucose-6-phosphate isomerase, and L-lactate dehydrogenase B chains, as well as altering the p53 and AKT expression. Zhu et al. [15] found in human gastric adenocarcinoma SGC-7901 cells that* Celastrus orbiculatus* suppressed TGF-*β*1-induced epithelial-mesenchymal transition by inhibiting HSP27 expression, and further investigation showed that the downregulation of HSP27 was associated with TNF-*α*-induced NF-*κ*B/Snail signaling pathway.

In breast cancer, proteomic analysis by Fang et al. [14] identified 12 differentially expressed proteins, of which the downregulated proteins TDP-43, SF2/ASF, and eIF3i, as well as upregulated proteins including 3-PGDH, ERP29, and platelet-activating factor acetylhydrolase IB subunit beta, positively contributed to the anticancer activity of Curcumin in human breast cancer MCF-7 cells. In addition, a study by Chou et al. [36] illustrated that berberine induced apoptosis of MCF-7 cells, and a link between ROS generation and cell death was identified using lysine- and cysteine-labeling 2D-DIGE combined with MS.

In leukemia K562 cells, Wei et al. [37] demonstrated that, after treatment with triterpenes from* Patrinia heterophylla*, 4 proteins were upregulated (aldolase A, glyceraldehyde-3-phosphate dehydrogenase, flavin reductase, and hemoglobin subunit) and 4 downregulated (heat shock protein 90 Alpha, eukaryotic translation initiation factor 5A, moesin, and tubulin). These proteins were associated with energy metabolism, oxidative stress, apoptosis, signal transduction, differential induction, and protein biosynthesis.

#### 4.1.2. Cardiocerebrovascular Diseases

In heart disease, Fan et al. [38] revealed that Shuanglong formula (SLF) induced autologous mesenchymal stem cells (MSCs) into cardiomyocyte-like cells, and 36 proteins, which functioned in cytoskeleton, cell tissue energy metabolism, and signal transduction, showed distinct differential expression patterns before and after SLF treatment. Feng et al. [39] clarified the signaling impact of salvianolic acid B (SB) in H9C2 cells using proteomic assay and bioinformatic analysis and found the signal cascade from EGFR to heat shock protein 27 (HSP27) and mitofilin might be the most important cascade that was affected by SB.

In Alzheimer's disease, Tao et al. [40] found that Huperzine A, from* Huperzia serrata*, protected N2a cells from amyloid *β*-induced cell death by decreasing the p53 protein levels.

In Parkinson's disease (PD), iTRAQ-based quantitative proteomics study [41] uncovered that, after treatment with extract of* Acanthopanax senticosus* Harms (EAS) in A53T-*α*-Syn transgenic SH-SY5Y cells, 16 out of 84 abnormally expressed proteins were altered. These proteins play roles mainly in formation of Lewy body, mitochondrial energy metabolism, protein synthesis, and apoptosis. Also in SH-SY5Y cells, Ramachandran et al. [42] discovered that Tianma promoted neuroregenerative signaling cascades by controlling chaperone proteins such as CALR, FKBP3/4, and HSP70/90, mobilizing a neuroprotective gene AIP5, and modulating RTN1/4, NCAM, PACSIN2, and PDLIM1/5 with various regenerative modalities and capacities related to neurosynaptic plasticity. In another proteomic research by Manavalan et al. [11], they proposed that Tianma promoted neuroregenerative processes in N2a cells by inhibiting stress-related proteins and mobilizing neuroprotective genes such as Nxn, Dbnl, Mobkl3, Clic4, Mki67, and Bax.

#### 4.1.3. Inflammatory Diseases

In inflammatory diseases, the proteome profiler array analyses [44] displayed that 8 cytokines were downregulated while 6 were upregulated by Bi-Qi capsule in lipopolysaccharide-stimulated RAW 264.7 macrophages. The same research group [45] also found that Zuojin pill inhibited the levels of inflammatory mediators such as inducible nitric oxide synthase (iNOS), cyclooxygenase-2 (COX-2), TNF-*α*, IL-6, and interleukin 1*β* (IL-1*β*) in lipopolysaccharide-stimulated RAW 264.7 mouse macrophages. Additionally, Jiang et al. [12] established that* Acanthopanax senticosus* extract (ASE) suppressed LPS-induced nitrosative stress in BV-2 cells, and proteomic quantitative analyses revealed that the levels of 17 proteins significantly changed in response to ASE.

In other diseases, by proteomic analysis, several important molecular mechanisms of TCM treatment have been found, such as Lithospermi radix on wound healing [46], isopsoralen on oxidative damage [47], Tao Hong Si Wu decoction (THSWD) on cerebral ischemia reperfusion injury [48], and Curcumin on postcataracts [49].

One may know that* in vitro* studies may not reflect the actual effects of TCM treatments, as* in vivo* body environment and the influencing factors are very complex and hard to control. To better understand the effective mechanisms of TCM treatments,* in vivo* animal models and tests are highly desired.

### 4.2. Evaluation of TCM Treatments* In Vivo*

Presently, the* in vivo* proteomic research involves mainly the rat and mouse models. Recent studies focused mostly on the following diseases: cardiocerebrovascular diseases, hepatorenal diseases, diabetes, and so on. In addition, proteomic technologies were also applied to study the effective mechanisms of acupuncture on diseases such as asthma. As shown in Table 2, the proteomics was applied to investigate the mechanisms of TCM treatments including CHF, CHM, and CHM compound* in vivo*.

#### 4.2.1. Cardiocerebrovascular Diseases

By proteomic analysis of heart tissues in rat ischemia/reperfusion (I/R) models, Jia et al. [50] confirmed that Dingxin recipe prevented ischemia/reperfusion-induced arrhythmias via upregulating prohibitin and suppressing inflammatory responses. In a rat model of myocardial infarction, Zhou et al. [51] established that Buyang Huanwu decoction (BYHWD) alleviated ventricular remodeling, which increased Bcl-2/Bax ratio and decreased caspase 3 activity via downregulating atrial natriuretic factor (ANF) while upregulating heat shock protein beta-6 (HSPB6) and peroxiredoxin-6 (PRDX6).

By the differential proteomic analysis in platelet samples of SD rats, Ma et al. [52] proposed that salvianolic acid B (SB) caused regulation of 20 proteins such as heat shock-related 70 kDa protein 2 (HSP70), LIM domain protein CLP-36, copine I, peroxiredoxin-2, coronin-1B, and cytoplasmic dynein intermediate chain 2C. Furthermore, SB bound with integrin *α*2*β*1 to regulate intracellular Ca^2+^ level and the levels of cytoskeleton-related protein coronin-1B and to affect cytoskeleton structure of platelets. In addition, Yue et al. [53] from the same studying group demonstrated that salvianolic acids (SA) and notoginsenoside (NG) showed both similarity and difference in their protein targets involved in cardioprotective effects.

Lo et al. [54] investigated the effect of* Uncaria rhynchophylla* (UR) on the differentially expressed proteins in SD rats with kainic acid- (KA-) induced epileptic seizures using a proteomic analysis and found that macrophage migration inhibitory factor (MIF) and cyclophilin A were underexpressed in frontal cortex by an average of 0.19- and 0.23-fold, respectively, suggesting that both MIF and cyclophilin A at least partially participated in the anticonvulsive effect of UR. Zhang et al. [55] explored the effective mechanisms of Yizhijiannao granule (YZJN) in treating Alzheimer's disease (AD) with proteomic tools, and the results indicated that YZJN regulated multiple protein expressions in entorhinal cortex tissues of SAMP8, suggesting that it had multitarget therapeutic action and the mechanism in treating AD is possibly via improving mitochondria function, antagonizing oxidation stress, preventing nerve cell apoptosis, and protecting neurons. Koh [56] has identified the proteins differentially expressed in cerebral cortexes of* Ginkgo biloba* extract- (EGb761-) treated rats in a middle cerebral artery occlusion model, and the results showed that EGb761 protected neuronal cells against ischemic brain injury through the specific up- and downmodulation of various proteins.

Manavalan et al. [43] found that* Gastrodia elata* (Tianma) affected synaptic plasticity and neurorestorative processes and thus might be a novel candidate agent for the treatment of neurodegenerative diseases by regulating the brain proteome. In detail, the long-term treatment with Tianma modulated the brain protein metabolism at the proteome level by downregulating the expressions of various proteins, such as Gnao1 and Dctn2, which are related to neuronal growth cone control and synaptic activities. Tianma treatment also induced the upregulation of molecular chaperons and proteins related to the misfolded protein response, such as Pacsin1 and Arf3 involved in Huntington's disease (HD).

#### 4.2.2. Hepatorenal Diseases

Using proteomics technologies and* in vivo* model, Shen et al. [57] investigated the effects of Yiguanjian decoction on rats with cirrhosis and found that increasing expression of proteins that were related to antioxidative stress such as Cu/Zn SOD and DJ-1 is probably the mechanism of Yiguanjian decoction in treating CCl4 induced cirrhosis. Xie et al. [58] proposed that the action mechanism of anti-liver fibrosis effect of Fuzheng Huayu (FZHY) may be due to modulation of proteins associated with metabolism and stress response, as well as myofibroblast activation, including aldehyde dehydrogenase, vimentin isoform (CRA_b), gamma-actin, vimentin, fructose-bisphosphate aldolase B, aldo-keto reductase, S-adenosyl homocysteine hydrolase isoform, and HSP90. Wang et al. [80] studied the effects of Isoline on mouse liver protein profile and showed that the liver samples from mice of Isoline group had about 13 differentially expressed proteins compared with the normal. These proteins may be involved in Isoline-induced liver injury, as 9 of them were involved in the process of oxidative stress or cellular energy metabolism.

Sun et al. [60] investigated mechanisms of the protective effects of* Salvia miltiorrhiza *polysaccharide (SMPS) against lipopolysaccharide- (LPS-) induced immunological liver injury (ILI) in Bacille Calmette-Guérin- (BCG-) primed mice, and the results showed that SMPS antagonized liver injury by upregulating the enzymes of the citric acid cycle, namely, malate dehydrogenase (MDH) and 2-oxoglutarate dehydrogenase complex, as well as inhibiting the NF-*κ*B activation by upregulation of PRDX6 and the subsequent attenuation of lipid peroxidation, iNOS expression, and inflammation.

Liu et al. [61] observed the effect of Tanshinone II A sodium sulfonate (TSNIIA-SS) on oxidative stress in mice and used two-dimensional electrophoresis (2DE) to find that TSNIIA-SS treatment not only improved DXR lesion but also regulated the expression of several proteins associated with the cytoskeleton, oxidative stress, and protein synthesis or degradation.

#### 4.2.3. Diabetes

In diabetes research, Guo and Xiong [62] observed changes of serum proteome in model rats treated with Granules of Eliminating Phlegm and Removing Blood Stasis (GEPRB). It suggested that 13 proteins changed in response to GEPRB* in vivo*, and these proteins may play key roles in the GEPRB treatment of diabetes deafness. Among them, 2 highly differentially expressed proteins apolipoprotein E (apoE) and C3 may be potential drug targets of GEPRB.

Shi et al. [63] investigated the effects of the Chinese medicine ZiBuPiYin recipe (ZBPYR) on the hippocampus in a rat model of diabetes-associated cognitive decline and found that 13 protein spots were altered between control and diabetes groups and 12 spots were changed between diabetes and DM/ZBPYR groups. Nine proteins were involved in energy metabolism, cytoskeleton regulation, and oxidative stress. The protein alterations observed in the diabetes group were ameliorated to varying degrees following ZBPYR treatment.

Another proteomic study of serum proteins in a type 2 diabetes mellitus (T2DM) rat model by Chinese traditional medicine Tianqijiangtang capsule was performed by Zhang et al. [64], and the distinct effect of T2DM on rat serum protein patterns included the downregulation of apolipoprotein E, apolipoprotein A-I, and Ig gamma-2A chain C region and upregulation of transthyretin (TTR), haptoglobin (Hp), serum amyloid P-component (SAP), and prothrombin. The majority of those protein levels were restored to those of healthy rats after Tianqijiangtang capsule treatment.

In other diseases, the proteomics was also applied to uncover the effective mechanisms of TCM treatments in* in vivo* models, such as chronic restraint stress-induced liver stagnancy syndrome rats [65], stressed rats treated with Wei Kangning [66], spontaneously hypertensive rats treated with CHM compound PingganQianyang [67], airway inflammatory mice treated with Xiao-Qing-Long-Tang [81], silica exposing rats treated with* Gymnadenia conopsea* alcohol extract [69], and anxious rats treated with polysaccharides extracted from Shudihuang [70]. In addition, the proteomics was also performed to study the effect of acupuncture on different diseases, such as the effects of acupuncture at Taixi acupoint (KI3) on kidney proteome [71], the nonspecific physiological background effects of acupuncture revealed by proteomic analysis in normal rats [72], and the proteomic analysis in acupuncture-treated rats with asthma onset [73].

In summary, from the* in vivo* models, using proteomics method, the researchers can successfully identify novel candidate proteins involved in the development of disease and define potential targets for TCM treatment. Further studies are required to investigate the exact role of these selected proteins and validate their potential as therapeutic targets.

## 5. Combination of Proteomics and Other “-Omics” Technologies in TCM Researches

Because of the complexity of TCM and the differential expression profiles of various biomolecules, that is, DNAs, RNAs, proteins, and metabolites, there are indeed numerous regulatory mechanisms and signaling pathways awaiting to be investigated for TCM research. Systematically integrating collected information from genomics, transcriptomics, proteomics, and metabolomics will greatly help explore the molecular mechanisms of TCM, as well as finding biomarkers or targets for future research and clinical practice.

Over the past few years, several researchers have combined the use of transcriptomics and proteomics in TCM researches. Using next-generation RNA sequencing and iTRAQ, in gastric cancer cell line AGS treated with TIIA, Lin et al. [35] characterized 16,603 unique transcripts and 102 proteins, which were involved in carbohydrate metabolism, cell cycle, apoptosis, DNA damage, and cytoskeleton reorganization. Intracellular ATP levels, the levels of glucose-6-phosphate isomerase, L-lactate dehydrogenase B chains, p53, and AKT were characterized to be associated with these changes. Moreover, proteomics and transcriptomic analysis coupled with pharmacological tests were employed to reveal the diversity of antithrombosis proteins from the medicinal insect,* Eupolyphaga sinensis*. By this approach, Wang et al. [82] found that serine proteases contained both plasmin- and plasminogen-activating-like activities, the excellent candidates for antithrombosis medicines.

Recently, an integrated proteomic and metabolomic study found that three principal components of Yin-Chen-Hao-Tang,* Artemisia annua* L.,* Gardenia jasminoides* Ellis, and* Rheum palmatum* L., contained major active ingredients 6,7-dimethylesculetin (D), geniposide (G), and rhein (R), respectively. Wang et al. [83] found that the DGR combination had a better therapeutic effect through intensifying dynamic changes in metabolic biomarkers, regulating target proteins, and activating both intrinsic and extrinsic pathways.

However, what needs to be emphasized is that, because of the high cost of “-omics” technologies, the studies combining proteomics with other “-omics” still remain of a small number, especially in TCM research, but it is a very promising approach since the combined “-omics” can provide much more information.

## 6. Prospects and Challenges

Recent technological advances in “-omics” including genomics, transcriptomics, proteomics, and metabolomics have helped cast light on the essence and molecular basis of TCM syndrome. High-throughput proteomics technologies assist the researchers to identify candidate proteins, which play key roles in TCM treatment response and toxicity. Subsequently, pharmacoproteomics emerges, seeking to characterize the molecules affecting the response to drugs in individual patients and helping target-based therapy. Furthermore, disease susceptibility proteins representing potential new drug targets could also be identified by pharmacoproteomics methods. All these novel approaches provide very useful tools for TCM drug discovery and individualized application of TCM therapy.

Proteomics can combine tightly with other technologies to better understand the essences of TCM [84]. For example, combining proteomics and bioinformatics to study protein signaling pathways and protein-drug interactions would be favorable to molecular evidence-based TCM research. Posttranslational modifications by small compounds, lipids, or even a group of chemicals can regulate the protein activity and its function. Proteomics could be applied to detect the posttranslational modifications and protein-protein interactions after TCM treatment. With the development of the latest proteomics techniques in TCM research, more and more researchers begin focusing on how proteomics can determine the mechanism of TCM treatment for various diseases including cancers, cardiocerebrovascular diseases, hepatorenal diseases, lung diseases, and diabetes. Proteomics could also be applied to determine the anticancer mechanisms of CHM compounds or CHM and CHF extracts. In addition, many researchers have paid more attention to proteomics studies based on treatment-TCM syndrome animal models. For example, Xie et al. focused on change of serum proteome in noxious Heat Blood Stasis syndrome treated by Radix [85]; Liao et al. performed an experimental study on proteomic analysis of gastric mucosa in chronic gastritis rats of Spleen-Stomach Damp-Heat syndrome treated by Sanren decoction [86]. These studies greatly help understand the molecular basis of TCM treatment based on syndrome differentiation.

However, every coin has two sides; proteomics technology has its limitation or disadvantage. Presently, the identified proteins from the proteomics technology are relatively fewer in comparison with the data from genomics and transcriptomics. The proteomics results are frequently instable and variable, which means that the reproducibility of proteomics data is poor. To obtain the ideal results, it is necessary to integrate all the proteomic technology such as 2DE, HPLC, MALDI-TOF-MS, SELDI-TOF-MS, MS/MS, iTRAQ, and the bioinformatics.

Furthermore, there are still many limitations for the application of proteomics in TCM research. For example, most studies have identified differentially expressed proteins among different TCM syndromes or TCM treatments. Few identified molecules were investigated in depth from the aspects of function and mechanism. Moreover, although many* in vivo* animal models associated with TCM syndromes have been established based on TCM theory, it remains unknown whether these models accurately simulate the real human environment and reflect human body conditions [87]. In addition, many CHM or CHF compounds are very complex and not stable because of the origin and production process; the proteomics results could not keep consistent, which affect greatly the further mechanism investigation of CHMs and CHFs.

Nowadays, proteomics bridges TCM and modern life sciences, greatly facilitates the quality evaluation and standardization of TCM, and promotes the modernization and internationalization of TCM. Although the application of proteomics in TCM research is full of challenges, it also provides very good opportunity.

## Figures and Tables

**Table 1:** Proteomics applied in the effective mechanisms of TCM treatments *in vitro*.

Diseases	CHF, CHM, and CHM compound	Targets or signaling pathways	Proteomics methods	References
Cancers				
Cervical carcinoma	9,11-Dehydroergosterol peroxide	Stathmin 1	MALDI-TOF MS/MS	[27]
Cervical carcinoma	Triterpenes	IL-17E, eIF5A, peroxiredoxin-2, and ubiquilin-2	2DE, MALDI-TOF MS/MS	[28]
Cervical carcinoma	Tanshinone IIA	Vimentin, Maspin, a- and b-tubulin, and GRP75	2DE, MALDI-TOF MS	[29]
Cervical carcinoma	Tanshinone IIA	Endoplasmic reticulum stress pathways	2DE, MALDI-TOF-TOF MS	[30]
Hepatocellular carcinoma	1,3,6,7-Tetrahydroxyxanthone	*β*-tubulin, 14-3-3*σ*, and P16	2DE, MALDI-TOF-MS, MS/MS	[31]
Hepatocellular carcinoma	1,3,5-Trihydroxyxanthone	HSP27	2DE, MALDI-TOF-TOF MS	[32]
Colorectal carcinoma	Baicalein	Peroxiredoxin-6	2DE, MALDI-TOF-TOF MS	[33]
Colon carcinoma	Triptolide	14-3-3*ξ*	2DE, MALDI-TOF-TOF MS	[34]
Gastric adenocarcinoma	*Celastrus orbiculatus*	HSP27, NF-*κ*B/Snail signal pathways	2DE, MALDI-TOF-TOF MS	[15]
Gastric adenocarcinoma	Tanshinone IIA	p53, AKT, G6PI, and LDHB	iTRAQ	[35]
Breast carcinoma	Curcumin	TDP-43, SF2/ASF, eIF3i, 3-PGDH, and ERP29	2DE, MALDI-TOF MS	[14]
Breast carcinoma	*Coptis chinensis* Franch.	ROS generation	2D-DIGE, MS	[36]
Leukemia	Triterpenes	Aldolase A, GAPDH, and HSP90-Alpha	2DE, MALDI-TOF-MS	[37]
Cardiocerebrovascular diseases				
Heart disease	Shuanglong formula	Energy metabolism	2DE	[38]
Cardiovascular disease	Salvianolic acid B	HSP27 and mitofilin	2DE, MALDI-TOF-MS/MS	[39]
Alzheimer's disease	Huperzine A	Trp53	LC-MS/MS	[40]
Parkinson's disease	*Acanthopanax senticosus* Harms	Lewy body, mitochondrial energy metabolism	iTRAQ	[41]
Neurodegenerative disorders	Tianma	CALR, FKBP3/4, HSP70/90, and AIP5	iTRAQ	[42]
Neurodegenerative disorders	Tianma	Nxn, Dbnl, Mobkl3, Clic4, Mki67, and Bax	iTRAQ	[43]
Inflammatory diseases				
Inflammatory diseases	Bi-qi capsule	iNOS, COX-2, TNF-*α*, IL-6, and IL-1*β*	Proteome profiler array	[44]
Inflammatory diseases	Zuojin pill	iNOS, COX-2, IL-6, IL-1*β*, TNF-*α*, and NF-*κ*B	Proteome profiler array	[45]
Neuroinflammation	*Acanthopanax senticosus* extract	Nitrosative stress pathway	2D-DIGE, LC-ESI-MS/MS	[12]
Other diseases				
Wound healing	Lithospermi radix	Antioxidant activity, antiapoptosis activity	2DE, LC-MS/MS	[46]
Oxidative damage	Isopsoralen	Proteins (*m/z* 6532 and *m/z* 6809)	SELDI-TOF-MS	[47]
Ischemia reperfusion injury	Tao Hong Si Wu decoction	Nrf2-mediated phase II enzymes	2DE, MALDI-TOF MS	[48]
Postcataracts	Curcumin	Proteins (*m/z* of 8093 and *m*/*z* 13767)	MS	[49]

**Table 2:** Proteomics applied in the effective mechanism of TCM treatment *in vivo*.

Diseases	CHF, CHM, CHM compound, and acupuncture	Targets or signaling pathways	Proteomics methods	References
Cardiocerebrovascular diseases				
Arrhythmias	Dingxin recipe	Prohibitin	2DE, MALDI-TOF MS	[50]
Ischemic myocardial injury	Buyang Huanwu decoction	Atrial natriuretic factor	2DE, MALDI-TOF MS	[51]
Cardiovascular disorders	Salvianolic acid B	Integrin *α*2*β*	2DE, MALDI-TOF MS/MS	[52]
Cardiovascular disorders	Salvianolic acids	Energy metabolism, lipid metabolism	2DE, MALDI-TOF MS/MS	[53]
Epileptic seizures	*Uncaria rhynchophylla*	MIF and cyclophilin A	2DE	[54]
Alzheimer's disease	Yizhijiannao granule	NADH dehydrogenase iron-sulfur protein 6	2DE, peptide mass fingerprint	[55]
Neurodegenerative diseases	*Ginkgo biloba* L. extracts	PPAP subunit Band CRMP2	2DE, MALDI-TOF MS	[56]
Cerebrovascular diseases	Tianma	Gnao1, Dctn2, Anxa5, Pacsin1, and Arf3	iTRAQ	[43]
Hepatorenal diseases				
Liver cirrhosis	Yiguanjian decoction	Cu/Zn SOD, DJ-1	2DE, MALDI-TOF-TOF MS	[57]
Liver fibrosis	Fuzheng Huayu	Vimentin	2DE/MS	[58]
Liver injury	Yin-Chen-Hao-Tang	Zinc finger protein 407, haptoglobin	RP-HPLC	[59]
Immunological liver injury	*Salvia miltiorrhiza*	PRDX6	2D-DIGE, MALDI-TOF MS	[60]
Nephropathy	Tanshinone IIA	Oxidative stress	2DE	[61]
Diabetes				
Diabetes	Granules	Apolipoprotein E (apoE) and C3	2DE, MALDI-TOF MS/MS	[62]
Type 2 diabetes mellitus	ZiBuPiYin recipe	DRP-2 and PDHE1*α*	Fluorescence-based DIGE, MS	[63]
Type 2 diabetes mellitus	Tianqijiangtang capsule	Haptoglobin, transthyretin, and prothrombin	2DE, MALDI-TOF-TOF/MS	[64]
Other diseases				
Gan stagnancy syndrome	—	TTR, aryl sulfotransferase	2DE, MALDI-TOF MS	[65]
Functional dyspepsia	Wei Kangning	Glutathione S-transferase, pi2	2DE, MALDI-TOF MS	[66]
Spontaneously hypertensive	Formula	HSP-27, annexin-A1, MFN-2, and Rho	2DE, MALDI-TOF MS	[67]
Allergic airway	Xiao-Qing-Long-Tang	Spectrin *α*2	2DE, MS, MS/MS	[68]
Silicosis	*Gymnadenia conopsea*	Cathepsin D precursor, peroxiredoxin-1	2DE, MALDI-TOF MS	[69]
Anxiety disorders	Polysaccharides	Beta-synuclein, DJ-1, and peroxiredoxin-2	2DE/MS	[70]
Normal	Acupuncture	NAD-isocitrate dehydrogenase	2DE, MALDI-TOF MS	[71]
Normal	Acupuncture	Local stimulus response, energy metabolism	2DE/MS	[72]
Asthma	Acupuncture	S100A8, RAGE, S100A11, and CC10	2DE, LC-MS/MS	[73]

## References

[1] Lu C.-L., Qv X.-Y., Jiang J.-G. (2010). Proteomics and syndrome of Chinese medicine. *Journal of Cellular and Molecular Medicine*.

[2] Lao Y., Wang X., Xu N., Zhang H., Xu H. (2014). Application of proteomics to determine the mechanism of action of traditional Chinese medicine remedies. *Journal of Ethnopharmacology*.

[3] Liu X., Guo D.-A. (2011). Application of proteomics in the mechanistic study of traditional Chinese medicine. *Biochemical Society Transactions*.

[4] Zhou H. G., Chen H. B., Zhou X. P. (2012). Proteomics is the important technology platform of Chinese medicine pathogenesis research. *Zhongguo Zhong Xi Yi Jie He Za Zhi*.

[5] Wei M., Liu Y.-P., Guo F.-H. (2011). Plasma proteomic analysis of patients with chronic hepatitis B of damp-heat retention in the middle-jiao syndrome. *ZhongguoZhong Xi Yi Jie He ZaZhi*.

[6] Liu Y., Liu P., Dai R. (2012). Analysis of plasma proteome from cases of the different traditional Chinese medicine syndromes in patients with chronic hepatitis B. *Journal of Pharmaceutical and Biomedical Analysis*.

[7] Song Y.-N., Zhang H., Guan Y. (2012). Classification of traditional Chinese medicine syndromes in patients with chronic hepatitis B by SELDI-based ProteinChip analysis. *Evidence-Based Complementary and Alternative Medicine*.

[8] Zhou Y.-W., Xu P.-C., Cheng Y. (2011). Basic pathogenesis of asthenia of healthy energy and blood stasis in liver cirrhosis studied by serum proteomics. *Zhongguo Zhong Xi Yi Jie He Za Zhi*.

[9] Hao Y. M., Hong M. C., Wang W. J. (2012). Study on proteins in urine of chronic renal failure patients of different TCM syndrome types. *Zhongguo Zhong Xi Yi Jie He Za Zhi*.

[10] Hao Y.-M., Hong M.-C., Wang W.-J. (2012). Study on correlated proteins in the urine of chronic renal failure patients of Chinese medicine damp syndrome based on SELDI-TOF-MS technique. *Zhongguo Zhong Xi Yi Jie He Za Zhi*.

[11] Manavalan A., Ramachandran U., Sundaramurthiet H. (2012). GastrodiaelataBlume (tianma) mobilizes neuro-protective capacities. *International Journal of Biochemistry and Molecular Biology*.

[12] Jiang T., Wang Z., Shenet R. (2015). Quantitative proteomics analysis for effect of acanthopanaxsenticosus extract on neuroinflammation. *Pakistan Journal of Pharmaceutical Sciences*.

[13] Cheng Z.-X., Liu B.-R., Qian X.-P. (2008). Proteomic analysis of anti-tumor effects by Rhizoma Paridis total saponin treatment in HepG2 cells. *Journal of Ethnopharmacology*.

[14] Fang H. Y., Chen S. B., Guo D. J., Pan S. Y., Yu Z. L. (2011). Proteomic identification of differentially expressed proteins in curcumin-treated MCF-7 cells. *Phytomedicine*.

[15] Zhu Y., Liu Y., Qian Y. (2014). Research on the efficacy of Celastrus Orbiculatus in suppressing TGF-*β*1-induced epithelial-mesenchymal transition by inhibiting HSP27 and TNF-*α*-induced NF-
*κ*B/Snail signaling pathway in human gastric adenocarcinoma. *BMC Complementary and Alternative Medicine*.

[16] Su S. B., Lu A., Li S., Jia W. (2012). Evidence-based ZHENG: a traditional Chinese medicine syndrome. *Evidence-Based Complementary and Alternative Medicine*.

[17] Chu Y.-G., Shi J., Hu Y.-H. (2009). Serum proteomes of hypertension patients with abundant phlegm-dampness. *Journal of Chinese Integrative Medicine*.

[18] Chu Y. G., Shi J., Hu Y. H. (2010). Serum proteomic study on hypertension patients with Gan-Dan damp-heat syndrome. *Zhongguo Zhong Xi Yi Jie He Za Zhi*.

[19] Song J.-N., Liu J.-L., Fang X.-Z. (2008). Relationship between plasma protein expression profiles and states of Zang-Fu organs in patients with phlegm or blood stagnation syndromes due to hyperlipidemia and atherosclerosis. *Zhong Xi Yi Jie He Xue Bao*.

[20] Zhao H.-H., Chen J.-X., Shi Q. (2010). Gel electrophoresis analysis on plasma differential protein in patients with unstable angina of blood-stasis pattern. *Zhongguo Zhong Xi Yi Jie He Za Zhi*.

[21] Wang Y., Chuo W.-J., Li C. (2013). Energy metabolism disorder and myocardial injury in chronic myocardial ischemia with Qi deficiency and blood stasis syndrome based on 2-DE proteomics. *Chinese Journal of Integrative Medicine*.

[22] Liu J., Li Y., Wei L. (2014). Screening and identification of potential biomarkers and establishment of the diagnostic serum proteomic model for the Traditional Chinese Medicine Syndromes of tuberculosis. *Journal of Ethnopharmacology*.

[23] Liu Z., Yu Z., Ouyang X., Du J., Lan X., Zhao M. (2012). Applied research on serum protein fingerprints for prediction of Qi deficiency syndrome and phlegm and blood stasis in patients with non-small cell lung cancer. *Journal of Traditional Chinese Medicine*.

[24] Wang Y.-Q., Li F.-F., Wang W.-J., Zhao L.-Y., Guo L., Wang H.-F. (2007). Serum proteomics study of chronic gastritis with dampness syndrome in traditional Chinese medicine. *Journal of Chinese Integrative Medicine*.

[25] Liu P., Zhang Y.-Y., Qiao J. (2007). Establishment and analysis of serum two-dimensional gel electrophoresis profiles of myasthenia gravis patients with spleen and kidney deficiency syndrome. *Journal of Chinese Integrative Medicine*.

[26] Lai M. S., Fan R. Q. (2010). Study on application of SELDI protein chip technique in diagnosis of systemic lupus erythematosus of yin deficiency caused internal heat syndrome. *Zhongguo Zhong Xi Yi Jie He Za Zhi*.

[27] Cui Y.-J., Guan S.-H., Feng L.-X. (2010). Cytotoxicity of 9,11-dehydroergosterol peroxide isolated from *Ganoderma lucidum* and its target-related proteins. *Natural Product Communications*.

[28] Yue Q.-X., Song X.-Y., Ma C. (2010). Effects of triterpenes from *Ganoderma lucidum* on protein expression profile of HeLa cells. *Phytomedicine*.

[29] Pan T.-L., Hung Y.-C., Wang P.-W. (2010). Functional proteomic and structural insights into molecular targets related to the growth inhibitory effect of tanshinone IIA on HeLa cells. *Proteomics*.

[30] Pan T.-L., Wang P.-W., Hung Y.-C., Huang C.-H., Rau K.-M. (2013). Proteomic analysis reveals tanshinone IIA enhances apoptosis of advanced cervix carcinoma CaSki cells through mitochondria intrinsic and endoplasmic reticulum stress pathways. *Proteomics*.

[31] Fu W.-M., Zhang J.-F., Wang H. (2012). Apoptosis induced by 1,3,6,7-tetrahydroxyxanthone in *Hepatocellular carcinoma* and proteomic analysis. *Apoptosis*.

[32] Fu W.-M., Zhang J.-F., Wang H. (2012). Heat shock protein 27 mediates the effect of 1,3,5-trihydroxy-13,13-dimethyl-2H-pyran [7,6-b] xanthone on mitochondrial apoptosis in hepatocellular carcinoma. *Journal of Proteomics*.

[33] Huang W.-S., Kuo Y.-H., Chin C.-C. (2012). Proteomic analysis of the effects of baicalein on colorectal cancer cells. *Proteomics*.

[34] Liu Y., Song F., Wu W. K. K. (2012). Triptolide inhibits colon cancer cell proliferation and induces cleavage and translocation of 14-3-3 epsilon. *Cell Biochemistry and Function*.

[35] Lin L. L., Hsia C. R., Hsu C. L., Huang H. C., Juan H. F. (2015). Integrating transcriptomics and proteomics to show that tanshinone IIA suppresses cell growth by blocking glucose metabolism in gastric cancer cells. *BMC Genomics*.

[36] Chou H.-C., Lu Y.-C., Cheng C.-S. (2012). Proteomic and redox-proteomic analysis of berberine-induced cytotoxicity in breast cancer cells. *Journal of Proteomics*.

[37] Wei D.-F., Wei Y.-X., Cheng W.-D. (2012). Proteomic analysis of the effect of triterpenes from *Patrinia heterophylla* on leukemia K562 cells. *Journal of Ethnopharmacology*.

[38] Fan X., Li X., Lv S., Wang Y., Zhao Y., Luo G. (2010). Comparative proteomics research on rat MSCs differentiation induced by Shuanglong Formula. *Journal of Ethnopharmacology*.

[39] Feng L.-X., Jing C.-J., Tang K.-L. (2011). Clarifying the signal network of salvianolic acid B using proteomic assay and bioinformatic analysis. *Proteomics*.

[40] Tao Y., Fang L., Yang Y. (2013). Quantitative proteomic analysis reveals the neuroprotective effects of huperzine A for amyloid beta treated neuroblastoma N2a cells. *Proteomics*.

[41] Li X.-Z., Zhang S.-N., Wang K.-X., Liu S.-M., Lu F. (2014). iTRAQ-based quantitative proteomics study on the neuroprotective effects of extract of *Acanthopanax senticosus* harm on SH-SY5Y cells overexpressing A53T mutant *α*-synuclein. *Neurochemistry International*.

[42] Ramachandran U., Manavalan A., Sundaramurthi H. (2012). Tianma modulates proteins with various neuro-regenerative modalities in differentiated human neuronal SH-SY5Y cells. *Neurochemistry International*.

[43] Manavalan A., Feng L., Sze S. K., Hu J.-M., Heese K. (2012). New insights into the brain protein metabolism of *Gastrodia elata*-treated rats by quantitative proteomics. *Journal of Proteomics*.

[44] Wang Q.-S., Cui Y.-L., Wang Y.-F., Chi W. (2011). Effects of compounds from bi-qi capsule on the expression of inflammatory mediators in lipopolysaccharide-stimulated RAW 264.7 macrophages. *Journal of Ethnopharmacology*.

[45] Wang Q.-S., Cui Y.-L., Dong T.-J., Zhang X.-F., Lin K.-M. (2012). Ethanol extract from a Chinese herbal formula, ‘Zuojin Pill’, inhibit the expression of inflammatory mediators in lipopolysaccharide-stimulated RAW 264.7 mouse macrophages. *Journal of Ethnopharmacology*.

[46] Hsiao C.-Y., Tsai T.-H., Chak K.-F. (2012). The molecular basis of wound healing processes induced by lithospermi radix: a proteomics and biochemical analysis. *Evidence-Based Complementary and Alternative Medicine*.

[47] Feng C.-Y., Huang X.-R., Qi M.-X. (2012). Mitochondrial proteomic analysis of isopsoralen protection against oxidative damage in human lens epithelial cells. *Chinese Journal of Integrative Medicine*.

[48] Qi H.-Y., Li L., Yu J. (2014). Proteomic identification of Nrf2-mediated phase II enzymes critical for protection of Tao Hong Si Wu decoction against oxygen glucose deprivation injury in PC12 cells. *Evidence-Based Complementary and Alternative Medicine*.

[49] Hu Y.-H., Huang X.-R., Qi M.-X., Hou B.-Y. (2012). Curcumin inhibits proliferation of human lens epithelial cells: a proteomic analysis. *Journal of Zhejiang University: Science B*.

[50] Jia Y.-H., Zhang Y.-X., Li L.-J. (2012). Dingxin recipe prevents ischemia/reperfusion-induced arrhythmias via up-regulating prohibitin and suppressing inflammatory responses. *Chinese Journal of Integrative Medicine*.

[51] Zhou Y. C., Liu B., Li Y. J. (2012). Effects of Buyang Huanwu decoction on ventricular remodeling and differential protein profile in a rat model of myocardial infarction. *Evidence-Based Complementary and Alternative Medicine*.

[52] Ma C., Yao Y., Yue Q.-X. (2011). Differential proteomic analysis of platelets suggested possible signal cascades network in platelets treated with salvianolic acid B. *PLoS ONE*.

[53] Yue Q.-X., Xie F.-B., Song X.-Y. (2012). Proteomic studies on protective effects of salvianolic acids, notoginsengnosides and combination of salvianolic acids and notoginsengnosides against cardiac ischemic-reperfusion injury. *Journal of Ethnopharmacology*.

[54] Lo W.-Y., Tsai F.-J., Liu C.-H. (2010). *Uncaria rhynchophylla* upregulates the expression of MIF and cyclophilin A in kainic acid-induced epilepsy rats: a proteomic analysis. *The American Journal of Chinese Medicine*.

[55] Zhang T., Dong K.-L., Li G.-C. (2010). Effect of yizhijiannao granule concentration fluid on the differential expression protein in entorhinal area tissue of senescence accelerated mouse P8. *Zhongguo Zhong Xi Yi Jie He Za Zhi*.

[56] Koh P.-O. (2011). Identification of proteins differentially expressed in cerebral cortexes of ginkgo biloba extract (EGb761)-treated rats in a middle cerebral artery occlusion model—a proteomics approach. *American Journal of Chinese Medicine*.

[57] Shen D.-Z., Tao Q., Du J.-X. (2010). Effects of Yiguanjian Decoction on liver cirrhosis formation:a differential proteomics study in rats. *Zhong Xi Yi Jie He Xue Bao*.

[58] Xie H., Tao Y., Lv J., Liu P., Liu C. (2013). Proteomic analysis of the effect of fuzhenghuayu recipe on fibrotic liver in rats. *Evidence-Based Complementary and Alternative Medicine*.

[59] Sun H., Zhang A., Yan G. (2013). Proteomics study on the hepatoprotective effects of traditional Chinese medicine formulae Yin-Chen-Hao-Tang by a combination of two-dimensional polyacrylamide gel electrophoresis and matrix-assisted laser desorption/ionization-time of flight mass spectrometry. *Journal of Pharmaceutical and Biomedical Analysis*.

[60] Sun X.-G., Fu X.-Q., Cai H.-B. (2011). Proteomic analysis of protective effects of polysaccharides from *Salvia miltiorrhiza* against immunological liver injury in mice. *Phytotherapy Research*.

[61] Liu X., Wang Y., Ma C. (2011). Proteomic assessment of tanshinone II A sodium sulfonate on doxorubicin induced nephropathy. *The American Journal of Chinese Medicine*.

[62] Guo H., Xiong D.-J. (2011). The proteomic research of the cure of experimental diabetes deafness by granules of eliminating phlegm and removing blood stasis. *Journal of Traditional Chinese Medicine*.

[63] Shi X., Lu X. G., Zhan L. B. (2011). The effects of the Chinese medicine ZiBu PiYin recipe on the hippocampus in a rat model of diabetes-associated cognitive decline: a proteomic analysis. *Diabetologia*.

[64] Zhang S.-X., Sun H., Sun W.-J., Jiao G.-Z., Wang X.-J. (2010). Proteomic study of serum proteins in a type 2 diabetes mellitus rat model by Chinese traditional medicine Tianqi Jiangtang Capsule administration. *Journal of Pharmaceutical and Biomedical Analysis*.

[65] Sun X.-G., Zhong X.-L., Liu Z.-F. (2010). Proteomic analysis of chronic restraint stress-induced Gan-stagnancy syndrome in rats. *Chinese Journal of Integrative Medicine*.

[66] Wei W., Li X., Hao J. (2011). Proteomic analysis of functional dyspepsia in stressed rats treated with traditional Chinese medicine ‘Wei Kangning’. *Journal of Gastroenterology and Hepatology*.

[67] Fan R., He F., Wang Y., Zhong G.-W., Li Y.-H. (2011). Changes of protein expression profile in vascular tissues of spontaneously hypertensive rats treated by a compound Chinese herbal medicine. *Zhong Xi Yi Jie He XueBao*.

[68] Nagai T., Nakao M., Shimizu Y. (2011). Proteomic analysis of anti-inflammatory effects of a kampo (Japanese Herbal) medicine ‘shoseiryuto (Xiao-Qing-Long-Tang)’ on airway inflammation in a mouse model. *Evidence-Based Complementary and Alternative Medicine*.

[69] Chen J. J., Chen L., Liu W., Wang S. X. (2012). Effects of Gymnadeniaconopse alcohol extract on early protein profiles in lung tissue of rats exposed to silica. *Zhonghua Lao Dong Wei Sheng Zhi Ye Bing Za Zhi*.

[70] Cui Y., Rong C., Wang J. (2013). Mechanism-based anti-anxiety effects of polysaccharides extracted from Shudihuang (*Radix Rehmanniae Preparata*) by two-dimensional electrophoresis analysis in rat hippocampus proteins. *Journal of Traditional Chinese Medicine*.

[71] Li C.-R., Cheng Z.-D., Zhang Z.-X. (2011). Effects of acupuncture at Taixi acupoint (KI3) on kidney proteome. *The American Journal of Chinese Medicine*.

[72] Xu Y.-D., Wang Y., Park G.-H. (2014). Non-specific physiological background effects of acupuncture revealed by proteomic analysis in normal rats. *BMC Complementary and Alternative Medicine*.

[73] Xu Y. D., Cui J. M., Wang Y. (2012). Proteomic analysis reveals the deregulation of inflammation-related proteins in acupuncture-treated rats with asthma onset. *Evidence-Based Complementary and Alternative Medicine*.

[74] Lin L.-L., Wang Y.-H., Lai C.-Y. (2012). Systems biology of meridians, acupoints, and Chinese herbs in disease. *Evidence-Based Complementary and Alternative Medicine*.

[75] Lian F., Wu H.-C., Sun Z.-G., Guo Y., Shi L., Xue M.-Y. (2014). Effects of Liuwei Dihuang Granule on the outcomes of *in vitro* fertilization pre-embryo transfer in infertility women with Kidney-yin deficiency syndrome and the proteome expressions in the follicular fluid. *Chinese Journal of Integrative Medicine*.

[76] Sun H., Zhang A., Yan G. (2013). Proteomics study on the hepatoprotective effects of traditional Chinese medicine formulae Yin-Chen-Hao-Tang by a combination of two-dimensional polyacrylamide gel electrophoresis and matrix-assisted laser desorption/ionization-time of flight mass spectrometry. *Journal of Pharmaceutical and Biomedical Analysis*.

[77] Pan S., Zhan X., Su X., Guo L., Lv L., Su B. (2011). Proteomic analysis of serum proteins in acute ischemic stroke patients treated with acupuncture. *Experimental Biology and Medicine*.

[78] Sun H., Zhang A. H., Yanet G. L. (2014). Acupuncture targeting and regulating multiple signaling pathways related to Zusanliacupoint using iTRAQ-based quantitative proteomic analysis. *Acupuncture and Related Therapies*.

[79] Qv X.-Y., Jiang J.-G., Piao J.-H. (2010). Pharmacodynamic studies of Chinese medicine at levels of whole animal, cell and molecular models. *Current Medicinal Chemistry*.

[80] Wang Z.-Y., Kang H., Ji L.-L. (2012). Proteomic characterization of the possible molecular targets of pyrrolizidine alkaloid isoline-induced hepatotoxicity. *Environmental Toxicology and Pharmacology*.

[81] Yamada H., Nagai T., Nakao M. (2011). Proteomic analysis of anti-inflammatory effects of a kampo (Japanese Herbal) medicine ‘shoseiryuto (Xiao-Qing-Long-Tang)’ on airway inflammation in a mouse model. *Evidence-Based Complementary and Alternative Medicine*.

[82] Wang Y., Yan H., Wang Y. (2012). Proteomics and transcriptome analysis coupled with pharmacological test reveals the diversity of anti-thrombosis proteins from the medicinal insect, *Eupolyphaga sinensis*. *Insect Biochemistry and Molecular Biology*.

[83] Wang X., Zhang A., Wang P. (2013). Metabolomics coupled with proteomics advancing drug discovery toward more agile development of targeted combination therapies. *Molecular and Cellular Proteomics*.

[84] Altelaar A. F. M., Munoz J., Heck A. J. R. (2013). Next-generation proteomics: towards an integrative view of proteome dynamics. *Nature Reviews Genetics*.

[85] Xie W. G., Ma X. C., Shao N. S. (2005). Preliminary study on change of serum proteome in noxious heat blood stasis syndrome treated by radix. *Zhongguo Zhong Xi Yi Jie He Za Zhi*.

[86] Liao S.-Y., Zeng J., Wang A.-Y., Chen J.-Y. (2013). Proteomic analysis of gastric mucosa in chronic gastritis rats of Pi-Wei damp-heat syndrome treated by sanren decoction: an experimental study. *Zhongguo Zhong Xi Yi Jie He Za Zhi*.

[87] Chen Z., Chen L.-Y., Wanget P., Dai H.-Y., Gao S., Wang K. (2012). Tumor microenvironment varies under different TCM *ZHENG* models and correlates with treatment response to herbal medicine. *Evidence-Based Complementary and Alternative Medicine*.

